# Therapeutic touch and therapeutic alliance in pediatric care and neonatology: An active inference framework

**DOI:** 10.3389/fped.2023.961075

**Published:** 2023-02-27

**Authors:** Zoe McParlin, Francesco Cerritelli, Andrea Manzotti, Karl J Friston, Jorge E Esteves

**Affiliations:** ^1^ Foundation COME Collaboration, Clinical-Based Human Research Department, Pescara, Italy; ^2^ Division of Neonatology, “V. Buzzi” Children's Hospital, ASST-FBF-Sacco, Milan, Italy; ^3^ Research Department, SOMA, Istituto Osteopatia Milano, Milan, Italy; ^4^ Wellcome Centre for Human Neuroimaging, Institute of Neurology, Queen Square, London, United Kingdom; ^5^ Malta ICOM Educational, Malta, Finland; ^6^ Research Department, University College of Osteopathy, Research Department, London, United Kingdom

**Keywords:** therapeutic alliance, active inference, affective touch, perinatal care, synchrony

## Abstract

Therapeutic affective touch has been recognized as essential for survival, nurturing supportive interpersonal interactions, accelerating recovery—including reducing hospitalisations, and promoting overall health and building robust therapeutic alliances. Through the lens of active inference, we present an integrative model, combining therapeutic touch and communication, to achieve biobehavioural synchrony. This model speaks to how the brain develops a generative model required for recovery, developing successful therapeutic alliances, and regulating allostasis within paediatric manual therapy. We apply active inference to explain the neurophysiological and behavioural mechanisms that underwrite the development and maintenance of synchronous relationships through touch. This paper foregrounds the crucial role of therapeutic touch in developing a solid therapeutic alliance, the clinical effectiveness of paediatric care, and triadic synchrony between health care practitioner, caregiver, and infant in a variety of clinical situations. We start by providing a brief overview of the significance and clinical role of touch in the development of social interactions in infants; facilitating a positive therapeutic alliance and restoring homeostasis through touch to allow a more efficient process of allostatic regulation. Moreover, we explain the role of CT tactile afferents in achieving positive clinical outcomes and updating prior beliefs. We then discuss how touch is implemented in treatment sessions to promote cooperative interactions in the clinic and facilitate theory of mind. This underwrites biobehavioural synchrony, epistemic trust, empathy, and the resolution of uncertainty. The ensuing framework is underpinned by a critical application of the active inference framework to the fields of pediatrics and neonatology.

## Introduction

Therapeutic touch has been shown to have positive physiological and psychological benefits for the pediatric population. This practice can be traced back to ancient China, where therapeutic massage was used on infants (1). Touch is considered to be a powerful sense in prenatal development, as it is the first sense to develop (2). Somatosensory receptors begin to develop at 4–7 weeks of gestation and fetuses show movement in response to touch on their lips at 7 weeks post-conception (3, 4). Brain responses to therapeutic touch begin between 11 and 36 days after birth, with the same activation patterns in the somatosensory and insular cortices observed in response to therapeutic touch in older individuals (5, 6).

Affective touch refers to the involvement of CT fibers in the tactile processing of sensory information within an emotional context (7). Low-velocity touch, often referred as affiliative, affective, pleasant or therapeutic, is characterized by slow, gentle stroking at skin temperature, typically 1 cm–10 cm/sec, which activates the unmyelinated C-Tactile afferent nerve fibers in the hairy skin. This type of touch is associated with the instinctual caress of a parent towards their infant and has been found to positively impact socio-emotional and cognitive development, as well as physiological and stress markers (1, 8–15). One well-known example of this is Kangaroo Care, where an infant's bare skin is placed on the bare chest of a caregiver, which has been shown to be beneficial in regulating an infant's physiology for over 40 years (16). In this paper, we will use the term therapeutic touch to link our arguments more effectively to paediatric care. Therapeutic touch has been shown to have a significant impact on the physiological and psychological well-being of neonatal and paediatric patients. Studies have found that therapeutic touch can positively regulate physiological markers such as epigenetics, neuroendocrine and stress markers (11, 12). These findings suggest that therapeutic touch plays a crucial role in the development of infants' socio-emotional and cognitive abilities, as well as their biobehavioural synchrony (1, 10, 13, 14). The positive effects of therapeutic touch on physiological and psychological markers further highlight the importance of incorporating this practice in healthcare settings, particularly for vulnerable populations such as infants.

The Social Baseline Theory posits that humans have evolved to be social creatures and that social interactions are essential for the efficient regulation of physiological and psychological processes (17, 18). Biobehavioral synchrony refers to the coordination of physiological and psychological processes during social interactions, such as the coordination of nonverbal behaviour, autonomic regulation, heart rhythms, brain-to-brain synchrony, and the release of neurotransmitters like oxytocin (19, 20). Research has shown that when these systems are sufficiently coupled, they exhibit similar dynamic neuronal structures, including matching activations in the parietal and frontal cortices, particularly during therapeutic touch (21, 22). It is suggested that caring therapeutic touch, which is both social and affective, may contribute to biobehavioural synchrony, affective physiological embodied predictions, and modulation of predictive homeostasis and nervous, immune, and neuroendocrine homeostatic regulation, which infants seem to outsource to caring adults (23, 24).

The therapeutic alliance, in the field of paediatrics and neonatology, often refers to a collaborative relationship between the child, parent, and practitioner. This alliance is essential for understanding family-centred care, ensuring child and parent satisfaction, and achieving positive clinical outcomes both in and out of the clinic. In this paper, the term “practitioner” refers to anyone providing manual or touch-based therapy, such as but not limited to physiotherapists, osteopaths, chiropractors, and massage therapists. A successful therapeutic alliance in paediatrics and neonatology encompasses the three core characteristics as described by Bordin (25); agreement on goals, tasks, and the development of a harmonious relationship between the therapist, patient, and their family. A strong therapeutic alliance is crucial for ensuring mutual trust, collaboration, and safety in all aspects of care (26). This is particularly important when dealing with a paediatric population, where the practitioner frequently encounters complex triadic relationships that depend on the interactions between the practitioner, caregiver, and patient.

Active inference is a theoretical framework that aims to understand sentient behavior by proposing that the brain actively creates generative predictive models of the external world based on the likelihood of incoming sensory inputs from both the individual's inner (interoceptive or proprioceptive) and outer (exteroceptive) environment. This creates the individual's own multisensory perception of the world, which may differ from reality (27, 28). It is important to note that the brain and nervous system prioritize balance and regulation in order to achieve a state of allostasis, or the expected physiological needs for survival (29–31). In the pediatric population, managing allostasis is closely connected to collaborative rapport and biobehavioral synchrony with others, as infants are unable to regulate their essential physiology, such as body temperature, without external help. Therapeutic touch has been proposed as an effective method for achieving allostatic regulation, physiological co-regulation, and embodied predictions related to social attachments, especially in infants with limited communication abilities (24, 32–34). The potential benefits of therapeutic touch may be partly attributed to its involvement of the insular cortex, which plays a fundamental role in attachment, alliance, and allostatic regulation, promoting rewarding synchronization, balance, and reducing pain (34, 35).

We propose that the use of therapeutic affective touch in paediatric manual therapy can facilitate the development of an ecological (therapeutic) niche and enhance the formation of a positive triadic collaborative relationship between the patient, caregiver, and therapist. Based on our recently published framework on the role of therapeutic touch in promoting biobehavioral synchrony within the clinical encounter (34), we suggest that touch is a crucial tool in facilitating homeostatic and allostatic regulation, ultimately enhancing the effectiveness of paediatric care.

## Application of active inference to touch and therapeutic alliance

Predictive coding postulates that the brain retains a sensitive balance of its internal generative model of how unobservable causes in the external and inner worlds generate sensations such as top-down predictions and bottom-up sensory inputs (28, 36). When there is a discrepancy between the expected or predicated sensory stimulation and the actual sensory input being experienced, the difference is referred to as a prediction error (37).

These prediction errors are used to guide and update prior or existing beliefs if deemed more precise, which are necessary to minimize this discrepancy that challenges and threatens their current generative model (36, 38–43). Precision reflects expectations of predictability, with increased precision weighting being elicited within a predictable and reliable setting (44). Significantly the temporoparietal and medial temporal lobes which are involved in both adapting, verifying, and reinforcing prior beliefs in specific contexts are also activated in affective touch (7, 45). Additionally minimizing prediction errors ensures self-regulation efficiency while also minimizing something called variational free energy, especially in ambiguous situations (46, 47). In this setting, free energy is the statistical measure of surprise or discrepancy associated with an unpredicted sensory stimulus in their current generative model (48). It has also been suggested that using additional sensory stimulation through therapeutic touch could minimize free energy. Research shows that comforting massage therapy for five days in preterm neonates significantly decreased energy expenditure to 1.8 Kcal/Kg/24 h (49). Similarly, individuals often collaborate to find the most efficient action through therapeutic touch. Intuitively, this amounts to minimizing the effort required to maintain homeostasis and accomplish the crucial task of growth and recovery, which is especially critical for infants who cannot usually achieve this on their own (18, 50, 51).

Significantly, the precision bestowed on a prediction error can be irrational and maladaptive, especially in chronic pain patients, physiologically and psychologically. Physiologically, the brain can alter the sensory states by increasing the precision to the selected sensory state allowing the belief to be updated at the higher levels of neural processing and correspondingly increasing the synaptic gain of secondary sensory and association cortices representing the associated prediction errors (52–54). Psychologically, this manifests as sensory attenuation (of sensory prediction errors) or selective attention (by increasing the precision of sensory prediction errors). This kind of precision weighting is mediated by modulating the gain of neural populations encoding prediction errors (55–59). The attenuation of sensory prediction errors can also be crucially applied to healthcare because it could determine whether an individual will actively ignore or pay attention to specific sensory data, especially in chronic pain (46). When someone is in chronic pain, it is not necessarily movement that causes the pain; instead, it is the inability to attenuate sensory evidence such as touch (31, 60). In other words, in an attempt to explain away somatosensory prediction errors that cannot be attenuated, the hypothesis of “I am in pain” is invoked, with all the accompanying pain-related behavior.

Similarly, this failure to attenuate sensory prediction errors could account for the self-stimulation and misalignment in dyadic interactions observed in severe autism; a syndrome thought to be characterized by a failure to attenuate sensory precision (61–65). It has been suggested that autism's lack of coherence between the brain's neural processing and touch awareness may contribute to these individuals' inability to recognize or explain therapeutic touch (66).

Moreover, it has been argued that, by using fMRI, it is possible to identify and minimize atypical responses and the processing of affective touch in children, allowing Therapist to observe and monitor the condition's development through therapeutic touch (67, 68). Notwithstanding this, certain forms of therapeutic massage appear to be effective in addressing and rehabilitating these touch-averse preferences in autistic children by reducing anxiety, increasing social communication and encouraging bonding (69). Therapeutic touch can be used to assist in focusing attention (i.e., optimizing precision gain) for specific prediction errors by modulating and deregulating top-down predictions initiated by touch-specific neurotransmitters in the hypothalamus-pituitary adrenocortical system (24, 70–75). Subsequently, generating a positive influence and makes sense of the physical world, helping the child become skilled in or mentalize the deployment of attention to current interoceptive signals; that they may struggle to do in conditions like autism. This may make it easier to nuance the precision afforded to higher-level prior beliefs to facilitate belief updating *via* attunement and synchrony rather than relying on previous expectations or priors (10, 41, 42, 76, 77). In a pediatric clinic setting, where there is often a triadic hierarchy, the practitioner is often perceived to have more authority and knowledge. This hierarchical model can influence the caregiver and child to rely on the practitioner's guidance in order to reduce their prediction errors and uncertainty, ultimately promoting homeostasis. In short, therapeutic touch enables individuals to recognize their maladaptive and overly precise beliefs and attempt to update or normalize them through a shared narrative of mutually predicted sensations; for example, learning that a fall does not necessarily mean they have broken something within a predictable and reliable setting (31, 44).

## The impact of touch on biobehavioural synchrony and infant physiology

Intentional social touch, particularly at speeds that activate CT fibers during social interactions, has been proposed to play a crucial role in attachment, communication, and regulation of physiology of individuals (78). Therapeutic touch interventions, such as massage, have been linked to various physiological benefits. Studies have found that these interventions can increase vagal tone, oxygen saturation, and dopamine levels, while decreasing cortisol, oxytocin, and stress levels (79–83). Additionally, it has been suggested that affective social touch can also contribute to establishing biobehavioral synchrony, which is known to assist in regulating an infant's physiology.

Affective CT-mediated touch is commonly used as an efficient medium for communication through social exchanges in various forms, such as a simple one-way exchange like a hand on a shoulder or dynamic reciprocal exchanges like a hug (84). This type of touch is considered an invaluable mode of communication, as it is used across cultures in various combinations to convey emotions through nonverbal exchanges (84). Social touch can effectively convey a wide range of emotions, including sadness, happiness, alertness, and calmness, and can contribute to the processing of facial emotions even if the touch is not directly targeted at the receiver (85, 86). Additionally, it is important to note that affective social touch is believed to have the same significance as direct gaze in four-month-old infants (87).

Social touch is often used in a reciprocal manner to communicate with others and build relationships. This is reflected in the way that dopaminergic reward systems respond to individuals touching each other. In fact, research has found that even one-month-old infants can potentially perceive pleasant touch and express motivation for future interactions, as demonstrated by somatosensory and insular activation reported by Tuulari et al. (5). Additionally, affective touch has been widely reported to reciprocally increase oxytocin levels in both individuals (88). However, in cases of post-natal depression where mothers engage in less touch, including affective, affectionate and breastfeeding, and in negative ways (e.g., rough pulling), infants may subsequently touch themselves more, potentially to compensate for this lack of touch (89, 90). This can lead to delays and negative consequences in socio-emotional and cognitive development, including higher risk of insecure attachment and bonding, as well as reduced cortisol levels (10, 14, 91). Furthermore, adults who experienced reduced touch early in life may not find affective touch more pleasant than other forms, suggesting that we may need to learn to enjoy and engage in touch (78, 92).

Affective touch plays a crucial role in facilitating mutual understanding, regulating physiology, and forming a robust therapeutic alliance between the practitioner, patient, and caregiver (82, 84, 87, 93, 94). Through the frequent use of affective touch as a mode of communication, it can help to develop behavioral synchrony, coupling, and a shared dynamic neural code between the practitioner, patient and their family (95). This can lead to an increase in rapport, trust, and mutual respect in the therapeutic relationship, allowing the caregiver to provide more supportive and autonomous care to the child's clinical needs (96, 97). Furthermore, incorporating family-centered care, which takes into consideration the values, attitudes, and context of the child's family in conjunction with the biomedical aspects of treatment, is regarded as best practice in pediatrics. The use of therapeutic affective touch in conjunction with family-centered care can help to attain patient satisfaction, biobehavioral synchrony, therapeutic alliance, and cooperative communication and improve both physical and psychological well-being for conditions such as juvenile idiopathic arthritis (96–100).

The application of biobehavioral synchrony in parent-infant interactions has been shown to result in a high level of synchronization, particularly with mother-infant heart rates, following therapeutic affective touch (80, 101). Research has also demonstrated that the oxytocin levels of mothers and fathers, following 15 min of play including affectionate and affective touch with their child, can predict the exact increase in oxytocin levels that the child will experience, due to the synchronization of the parents' hormones and behavior within the triadic family unit (102). At three months of age, infants are able to initiate synchronization of coordinated affectionate affective touch and body movements with their parents to achieve parasympathetic co-regulation, owing to the increased vagal tone acquired during social engagement (103, 104). Furthermore, therapeutic touch has been shown to assist in the physiological adaptation and regulation of the autonomic system following birth trauma or daily life stresses, through skin-to-skin and affective touch immediately following birth, and when being carried while the parent walks, increasing cardio-respiratory coupling parameters such as heart rate variability and body temperature, while decreasing crying and body movements (105–107). The available evidence suggests that biobehavioral synchrony, established through affectionate affective touch, can assist young infants in developing a blueprint for achieving and creating generative models for their physiological needs (103, 104).

The significance of parental touch in the development and well-being of infants has been well-documented in various studies. As proposed by Meaney et al. (108), parental touch can signal to the infant that their environment is safe and supportive, allowing them to adapt and thrive in that environment. This theory is supported by research that has shown how caregiving touch evokes physiological and epigenetic changes that decrease stress responses and enable increased infant learning and exploration (78, 109). Furthermore, a study by Feldman (110) found that premature infants who received skin-to-skin contact exhibited better exploratory behaviours at 6 months of age, highlighting the positive impact of parental touch on infant development. Overall, it is clear that parental touch plays a crucial role in the formation of a secure attachment, the regulation of physiology, and the development of cognitive and socio-emotional skills in infants.

Consequently, long-term infant-caregiver biobehavioral synchrony can be observed, with preterm infants receiving Kangaroo care showing greater synchrony than preterm infants receiving incubator care. However, this synchrony is less pronounced compared to that observed in full-term infants (33). But, by adulthood, adult child-parent synchrony is equal between preterm infants who received kangaroo care and full-term infants, highlighting the lasting impact of therapeutic touch on the long-term synchrony between mother and infant (33).

## Touch and allostatic regulation in paediatrics and neonatology

The concept of allostasis, which refers to the continuous adjustment of an individual's internal physiological or behavioural state through a complex neuro-humoral system, is central to understanding how therapeutic touch can aid in the regulation of an individual's physiology (33, 111). Social affiliation, whether intentional or not, can assist in co-regulating another individual and strengthen their relationship, as they subconsciously recognize that the other individual plays a crucial role in regulating their allostasis and fulfilling their interoceptive predictions more efficiently (23, 112). Affective parental touch, such as stroking, can aid in aspects of cognitive, metacognitive, and embodiment processing, particularly in infants whose brains are still too immature to efficiently regulate themselves (24, 110, 113). This type of touch is considered an embodiment ostensive cue, serving the evolutionary need to assist, change or delay another individual's interoceptive needs by creating opportunities or epistemic gains, such as facial recognition (86, 104). Overall, therapeutic touch can contribute to allostatic regulation by providing a sensory stimulus to create, modify, or update existing beliefs.

Feldman's 2020 study on “kangaroo care” investigated the long-term effects of 2 weeks of skin-to-skin contact in premature, low birthweight infants compared to maternal separation (110). The study found improved autonomic function, orientation, and information processing in the neonatal period, consistent with other research suggesting that parental touch contributes to co-regulation and synchronization between an infant and a caregiver, potentially reducing cortisol and respiratory rate, regulating peripheral and core temperatures in premature or newborn infants, and addressing a variety of critical physiological vulnerabilities in neonates (114–117). The study also found continued improvements in physiological and cognitive regulation, arousal, and social interactions up to 2 years old and at 10 years old, the infants showed more adaptive autonomic nervous systems in terms of cardiac and stress regulation, cognitive flexibility, and sleep patterns compared to the separated infants. This may be partly due to synchronization with a caregiver, helping to minimize prediction errors and regulate allostasis, especially during times of distress or uncertainty in the external environment, which could put additional strain on their allostatic regulation (23, 118, 119). Additionally, socio-affective regulation elicited by touch can result in beneficial changes in inflammation, immunity, stress, and allostatic load, as well as the effort required to maintain homeostasis in critically ill, premature, or multifactorial medical conditions (18, 51, 120, 121).

Feldman (2022) also found that individuals who received “kangaroo care” (skin-to-skin contact) in their infancy exhibited more adaptable empathic responses in young adulthood (18–20 years). The increased synchrony from early attachment experiences is believed to have provided a pathway between the amygdala, insular and temporal pole, allowing for better recognition and understanding of others' affective states, thereby preparing them for adult social life (110). Furthermore, the combination of insular projections and specific temporo-social computations exhibited in social touch interactions allows for the creation of joint predictive models, which can aid in understanding and predicting the behaviour of others (33, 59).

Therapeutic touch, such as massage, has been shown to have a positive impact on individuals experiencing allostatic overload by promoting relaxation and reducing stress levels. Studies have found that therapeutic touch can increase vagal tone, oxygen saturation, and dopamine levels, while decreasing cortisol, oxytocin, and stress levels (79–83). Additionally, therapeutic touch is believed to symbolize the physical unity of the therapeutic alliance, indicating that the practitioner is willing to share their resources and work together with the patient to resolve clinical symptoms (18). Furthermore, research suggests that receiving therapeutic touch is more effective than self-care at mitigating and regulating the effects of physical and emotional stress (110, 122).

This theory, paired with the social concept of “mommy” suggested by Atzil and colleagues (2018), can be applied in a clinical setting (23). “Mommy” represents a computational predictive model that uses previous experiences of exteroceptive information about a caregiver associated with interoceptive information about allostasis, with the brain subsequently making predictions for allostatic regulation based on social information or vice versa. Patients experiencing symptomatic distress will have gathered interoceptive information, such as pain from inflammatory mediators, to infer that they are facing allostatic overload. It has been suggested that patients may have developed exteroceptive beliefs that practitioners generally have expertise and success in promoting recovery, or from previous experiences with practitioners, to help them infer that treatment, including manual therapeutic touch, will contribute to their recovery. Additionally, it has been suggested that patients can remember a particular practitioner's distinctive touch or feel they understand their specific pathology from previous painful episodes. By examining, treating, reassuring, and assisting the patient in reconnecting and learning about their injury through tactile feedback, practitioners can help reduce allostatic overload, while also assisting in developing a successful therapeutic alliance and interpersonal relationship (118, 123).

Despite some promising clinical results for therapeutic affective touch, its effectiveness remains uncertain. Practitioners recognize that their outcomes are influenced by various sensory modalities, including non-verbal and verbal communication, which can impact a patient's recovery (31, 124). It is possible that expectation-associated placebo effects contribute to reduced pain reported in sham treatments, particularly in chronic pain, through non-conscious Bayesian biases (31). During a therapeutic intervention, whether sham or standard, the body's regulatory system makes inferences about internal and external states to minimize prediction errors and regulate allostasis (125). Therapeutic affective touch provides the individual with evidence to update their existing beliefs. Implementing therapeutic touch within a supportive environment can help patients infer that the touch will aid in resolving their symptoms.

## Touch exploration, developing and adapting priors through coupled action-perception cycles

Affective touch, particularly in social situations, has been shown to play a role in direct affective and socio-affective regulation through cognitive and embodied processes, including behavioural synchrony, leading to physiological co-regulation (24). Affective and therapeutic touch can aid in recreating biofeedback loops that enhance the salience and learning of allostatic regulation in specific contexts. Early social contact has been linked to the development of positive evolutionary beliefs and physiological mechanisms, such as dopaminergic and opioidergic pathways, that promote and enjoy social touch and attachments (126). Furthermore, the perception of affective touch is not only associated with attachment patterns but also with the intent of the individual experiencing pain modulation from touch (127). Caregiving affective touch, including touch used in daily caregiving tasks such as feeding, washing, and transporting, has been found to be soothing and linked to embodied mentalization of interoception. This suggests that essential daily caregiving tasks not only promote attachment but also help modulate infant physiology and internal beliefs, shaping future allostatic predictions and generative models (24, 51). Caregiving affective touch can secure interoceptive predictions, reducing prediction errors and providing calming effects, reducing infant distress (128). An example of this would be a caregiver who picks up a crying infant to feed them, fulfilling their allostatic nutritional needs while providing social support and comfort to reduce distress. Affective touch in social environments has been suggested to provide external opportunities for individuals to learn how to self-regulate their own allostasis in specific social and physical situations (112, 129).

Stroking and other forms of affective touch activate CT fibres thus activating the central hubs and pathways for oxytocinergic modulation, triggering the release of oxytocin. Oxytocin plays a significant role in both social attachments and providing allostatic regulation through the modulation and regulation of social and non-social behaviours within fluctuating social contexts (130, 131). Additionally, it has been suggested that social affective touch in childhood may optimize an adult's ability to utilize oxytocin when dealing with stress as adults. Affective touch, by triggering the release of oxytocin, allows individuals to expect and perceive touch as being pleasant, therefore motivating, stress-regulating and increasing the sense of safety in regard to their cognitive and external environment (109, 132). This sense of safety has been suggested as the theoretical mechanism behind why Tanaka and colleagues found that infants that received more social touch, particularly affectionate affective touch, had enhanced object exploration, both in reduced hesitancy to explore objects and spent more time exploring them than infants that received less touch. Additionally, the infants may be motivated to explore their external environment to receive potential rewards with less risk, anxiety, or punishment (133). This can be observed in infants who intuitively adapt their sucking mechanism to different textures and temperatures, such as from a mother's nipple to a bottle plastic nipple, to meet their nutritional requirements for survival, with body temperature creating the most optimal conditions for the activation of C-tactile afferents (134–136). Body movements and tactile sensations are crucial for exploratory perceptual learning of (the consequences of) foundational actions, with the changing proprioceptive information helping reveal the object's pure tactile properties (137).

The intention behind therapeutic affective touch may significantly impact how practitioners establish a therapeutic alliance through their portrayal of therapeutic touch, implying an enactive component to therapeutic touch (124). By using therapeutic touch that activates CT fibres and triggers the release of oxytocin, practitioners can communicate and reassure the patient with a sense of safety about their clinical condition, as reflected by an increase in heart rate variability of preterm infants after experiencing CT-activating stroking, which helps regulate their parasympathetic nervous system (138). Additionally, therapeutic affective touch activates the brain to develop sensitivity or trust to specific affective stimuli as a modality for acquiring precise, safe, and newsworthy sensory information, resulting in an increased sense of self-awareness (139). Even at 14 weeks gestation, twins direct their movements toward their co-twin to explore, communicate, and differentiate self from others, thereby effectively developing self-awareness and early social affective touch (140, 141). It is important to note that while exploration is predominantly detected through hedonic AB fibres than CT fibres, research by Sailer and Ackerley (92) concluded that individuals with reduced touch exposure interpret hedonic touch differently from controls, controversially perceiving it as more pleasant than affective touch, suggesting that the normal perception of gentle dynamic touch can in some cases overlap with other forms of touch. It has also been argued that a significant proportion of infant exploration occurs within social situations where the use of affective social touch is more prominent and environmental safety plays a bigger role.

## Coupled action-perception cycles within treatment

The process of aligning one's beliefs and actions with those of others to create a shared understanding is known as the canonical loop of coupled action-perception cycles (59). In clinical settings, incorporating therapeutic affective touch into social interactions between the practitioner, patient, and their families can enhance alignment and synchrony at multiple levels (142). This allows for the use of action-perception loops to infer and synchronize mental states through touch as a means of communication. Additionally, therapeutic affective touch can have a significant impact when treating infants, as it allows for the constant inference of mental states that may not be fully conveyed through verbal explanations and reassurance (143).

It is important to note that in triadic relationships, personal biases are often more pronounced and influential than in dyadic interactions and are typically expressed through physical social affective touch and social gaze (144). Additionally, factors such as gender can affect the way in which caregivers display and interpret social touch (145). For example, compared to mother-infant interactions which are characterized by gradual positive effects centred around intricate facial cues, father-infant interactions are typically characterized by high, abrupt arousal with numerous peaks centred on more physical play and games (145, 146). Similarly, it is suggested that similar mechanisms occur in therapist-infant interactions, as they are more likely to resemble a combination of affective slow movements as well as physical object-focus play, with therapeutic affective touch as the primary mode of communication.

Another important aspect to consider is that affective touch, while being a fundamental building block for inferring an infant's internal state during caregiving tasks, is only one aspect of multisensory interactions that also includes visual, auditory, and olfactory stimuli. Within a clinical setting, while affective therapeutic touch is often the primary mode of communication during sessions, other sensory stimuli also play a role. Research has shown that therapeutic touch, compared to verbal reassurance, can significantly reduce stress by affecting physiological, epigenetic, and neuroendocrine functions, thus demonstrating its superiority in providing social support, particularly in times of pain or distress (108, 147, 148).

We propose that the mechanism of developing neural synchrony in a clinical setting is heavily influenced and enhanced by therapeutic touch. This can be explained by expanding and combining the models proposed by Shamay-Tsoory and Eisenberg (122) and Bilek and co-workers (93). During consultations, the practitioner begins by evaluating the patient's clinical presentation in the context of allostatic overload and disrupted homeostasis, which establishes the diagnostic evaluation. Subsequently, the practitioner adjusts their thinking to reflect this decision and accompanying mental state. This mental state will manifest itself in the practitioner's behaviour, such as their facial expressions and how they conduct their hands-on evaluation and therapeutic intervention. The patient and caregiver will observe and sense these behaviours, often through affective touch, ultimately inferring the practitioner's mental states. Subsequently, their actions will influence the interpretation and adaptation of their predictions and biases if the recipient determines the suggested theory is more precise. Due to the practitioner's expertise and potentially therapeutic affective touch through the triggering of oxytocin, their diagnosis is likely to be regarded as more precise and trustworthy than the patient's or caregiver's assessment of the source of the pain. Moreover, the patient and caregivers will be able to assess rapport and the robustness of the therapeutic alliance through touch and other non-verbal cues (96). Anticipating how the patient and caregiver will react is accomplished through top-down predictions made prior to the response occurring (149). The practitioner's constant inference about the patient's mental states enables them to adapt their use of therapeutic affective touch, helping to create opportunities for the patient to modify and mitigate their predictions to increase the success of the therapeutic intervention. The exteroceptive cues obtained by the practitioner through therapeutic touch, in conjunction with subsequent cognitive and affective reassurance and empathy, is transferred to the insula during treatment and has been proposed to integrate into the development of new and updated priors to prepare for and regulate future allostatic disturbances (150, 151). As a result, the neural coupling, synchronization, and alignment that occurs in the default, higher-level temporal and parietal regions during this process will aid in the prediction of future interactions like this one (152). Arguably, this process will aid in the positive adaptation of maladaptive beliefs and learning in paediatric patients and their caregivers.

## The functional anatomy of touch and empathy within a clinical encounter

Empathy is an essential element of establishing a robust therapeutic alliance, enhancing patient satisfaction, clinical outcomes, reassurance, compliance, and assisting in reducing distress (153, 154). Empathy is central for practitioners to understand, acknowledge, and appreciate the patient's symptoms and difficulties on the road to recovery to foster trust and a strong relationship (25). The more empathy and respect the practitioner demonstrate to the patient and family, the stronger the therapeutic alliance is likely to be within triadic interactions, thereby increasing the clinical effectiveness of the intervention (98, 122).

The capacity to empathise with another stems from prior biobehavioural synchronous relationships, experiences, and predictions about social interactions involving emotion regulation, stress management, and cognitive control (33, 104). Through experience-dependent plasticity in the temporal, prefrontal, parietal, amgydala and insula regions it has been suggested that therapeutic touch in childhood enhances the ability to accurately recognize other people's emotions allowing young adults to empathise with others (33). These adaptations occur due to the integration of subcortical, paralimbic, and cortical structures that combine bottom-up recognition of others' emotions with top-down mentalization and emotional regulation to display an empathic response (155). Moreover, the amygdala and insula play an essential role in empathy and socio-emotional regulation and the development of mother-infant synchrony, which frequently indicates an individual's future ability and level of empathy (33, 156, 157). This is reflected in the fact that children who receive affective touch consistently throughout their childhood are more sensitive to the emotions of others as adolescents and thus become more empathic and likely to build better therapeutic alliances in the future.

**Figure 1:**
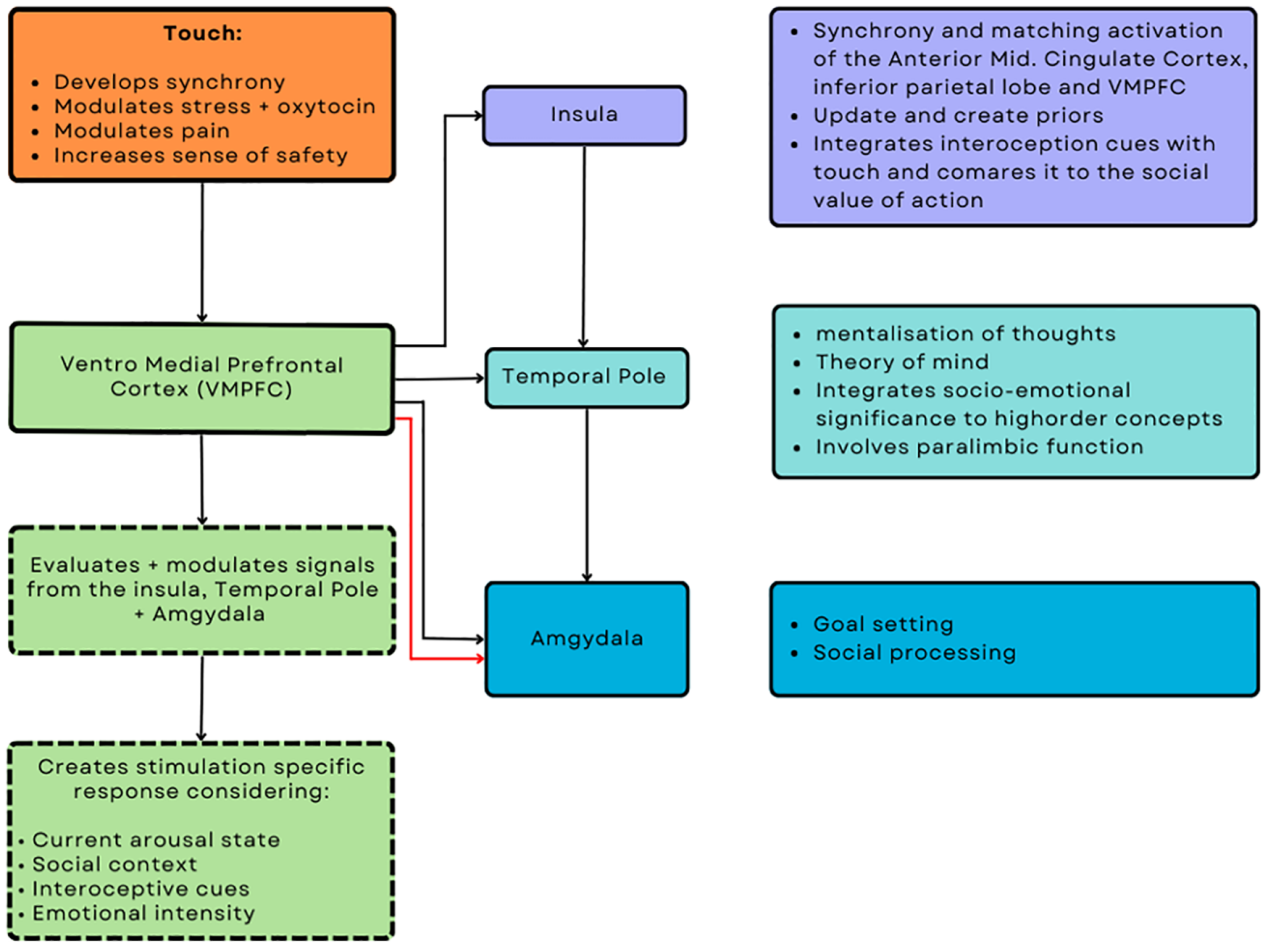
Putative neuromodulatory effects of touch from an interoceptive and interpersonal inference perspective.

Ulmer-Yanvi and colleagues (33) proposed that skin-to-skin contact occurring from infancy contributes to establishing integrative interoceptive cues that develop the complex mechanisms needed to establish empathy. Research suggests that the insula, during therapeutic affective touch, assists in updating and creating prior beliefs *via* a mechanism of integrating interoceptive cues and social values, which also correlates to the neuronal activity when experiencing pain or displaying empathy (158, 159). Additionally, the insula projects to the temporal lobe, which is involved in mentalization and theory of mind. The temporal lobe further integrates salient socio-emotional sensory inputs into higher-order concepts through its role as a paralimbic region, before projecting to the amygdala (160). The amygdala plays a role in emotional and social processing and goal setting before developing a response to stimuli and emotional states, which is then modulated by the ventral medial prefrontal cortex (VMPFC). The VMPFC modulates the overall process once more due to its significant reciprocal connections with both the insula and temporal lobe. The VMPFC generates affect-specific empathic responses by considering current arousal, social, internal states, and emotional intensity. Furthermore, the VMPFC plays a crucial role in developing synchrony, the modulation of oxytocin-related stress, the sense of safety, and pain, all of which can be modulated through therapeutic touch and are integral to therapeutic care. This neurological framework of touch and its subsequent biobehavioural synchrony within a clinical setting corresponds to activation, particularly in the anterior mid-cingulate cortex, inferior parietal lobes, and anterior insula, enabling individuals to demonstrate empathic qualities essential for the formation of a successful alliance (21, 161). Moreover, the shared emotions experienced during synchrony initiated by therapeutic affective touch allows for more efficient communication and precise inference of emotions and actions by reinforcing and increasing their relationship, attachment, feelings, and priors. Unsurprising that empathy is subsequently regarded as a crucial aspect of a superior practitioner and establishing a robust alliance, given its interconnections with attachment and synchrony, as well as its role in establishing a shared narrative, experience, emotional transfer, and reinforcing the belief that everyone is the same (see Figure 1).

## The clinical role of touch in overcoming uncertainty through creating a safe clinical environment

Bowlby (1988) proposed that an attachment figure can provide another individual with a strong sense of security (162). Therapeutic touch, which includes slow movements, skin-to-skin contact, and carrying, is a type of “comfort contact” that can help the recipient feel at ease, supported, and secure in the presence of another person, while also promoting stress regulation and healthy physiological and psychological development (51, 163, 164). The attachment bond between a mother and infant, which is often strengthened and initiated through tactile afferent touch, also underpins an infant's safety and comfort (165, 166). Moreover, it has been demonstrated that an effective alliance centred around treatment using therapeutic affective touch improves the child's safety and the child's and parent's well-being (98). Therapeutic touch can serve as a “secure base” by instilling a sense of social support and security in the clinical setting, thereby enhancing the robustness of the alliance and relationship with the patient (167, 168). Additionally, therapeutic affective touch is associated with decreased physiological arousal, bidirectional physiological regulation, and decreased pain thresholds (163). The relationship between the toucher and receiver seems to play a role in how touch is modulated and interpreted, with a closer relationship allowing for a higher variety of locations, slower tempo, and more intimate gestures (169–171). This is reflected in the responses observed in the insula to slow therapeutic touch, which is more significant than fast touch, observed in both 2-month-old and 2-year-old infants (68).

Additionally, the social content, including the identity and relationship of the toucher, seems to be more critical than other external factors (169). However, it is important to note that Pirazzoli et al. (2019) suggested that younger infants at 5 months old may need a more robust multisensory social experience than touch alone to identify touch as affective (172). Moreover, it has been suggested that the sense of safety often felt because of affective social touch loses its sensitivity as the individual gets older, partly as the attention bias for social threat decreases with age, correlating with the common desire to be independent from their parents (109, 173).

The sense of security achieved in the pediatric clinical setting has been attributed in part to the significant use of therapeutic affective touch, which has been associated with decreased physiological arousal, bidirectional physiological regulation, and decreased pain thresholds (163). Therapeutic affective touch can physically represent increased social support, which contributes to a sense of security by assisting in the modulation of pain, detecting potentially noxious stimuli, and augmenting the precision of non-noxious stimuli. These changes are attributed to increased activation of the prefrontal cortex, which is involved in pain modulation, sense of safety, and neural synchrony (131, 174). Therapeutic touch involving C-Tactile afferents modulates nociceptive signaling, preventing signals from reaching the brain and mediating nociceptive input at a subcortical level (175). The activation of CT fibers has been linked to having an anti-nociceptive role, suppressing C-nociceptive activity, weakening the temporal summation of second pain, and mediation of allodynia (176–178). Moreover, combined with emotional support and activation of the reward system triggered by comforting affective therapeutic touch, patients can have an increased tolerance for noxious nociceptive signals and thus an increased sense of security in knowing that their allostatic and emotional regulatory needs will be met (Figure 1). Additionally, this is reflected in chronic pediatric patients who also improve following massage therapy for musculoskeletal symptoms such as muscle spasms, tension, oedema, pain, distress, and mood disorders (179).

## Conclusion

**Figure 2:**
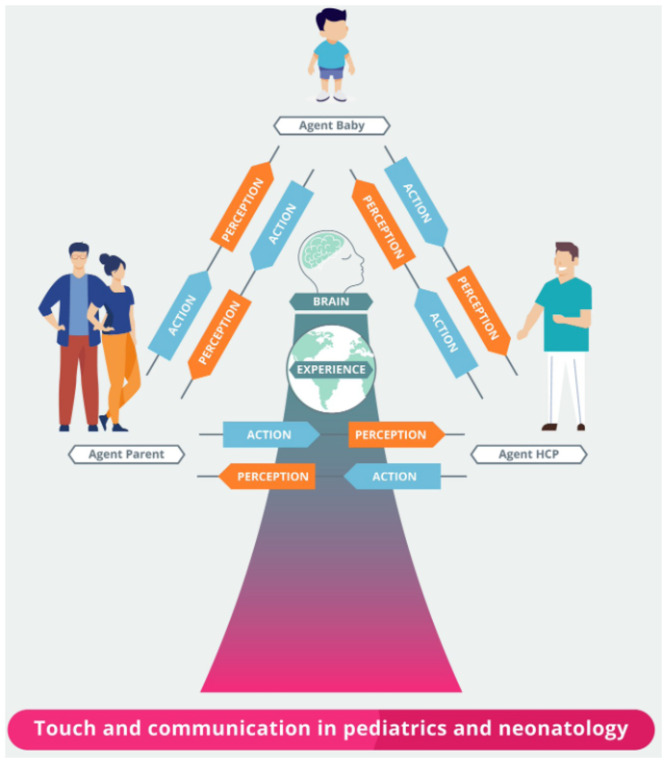
Active inference in paediatric care and neonatology. HCP: Health Care Provider.

**Figure 3:**
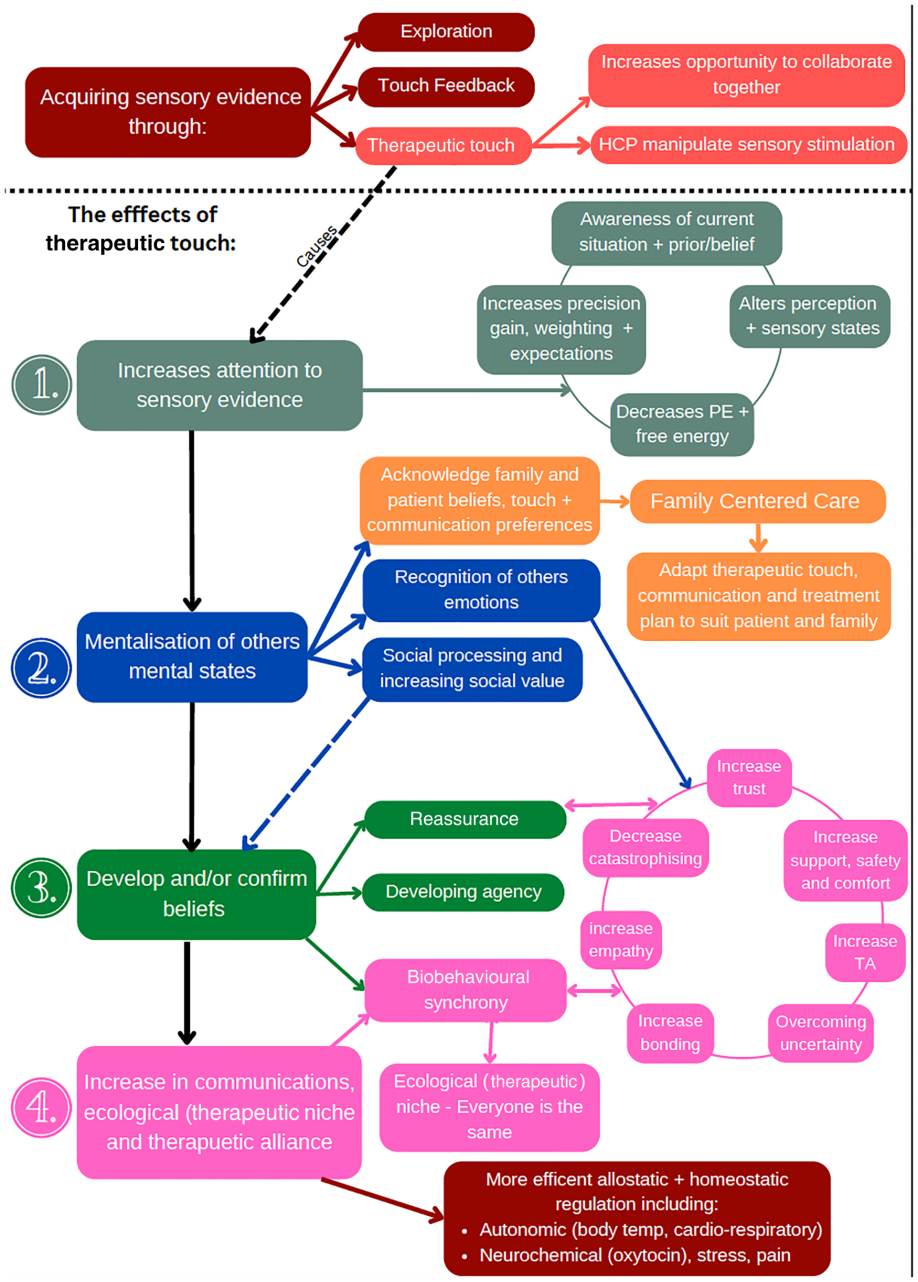
A map of the different processes involved in therapeutic touch.

Therapeutic touch plays a critical role in regulating allostasis and promoting homeostasis, developing socioemotional and cognitive systems, and achieving a robust therapeutic alliance in the paediatric population. Our research presents an integrative (active inference) model that helps explain the mechanisms by which therapeutic touch works. Through the use of this model, we propose that therapeutic touch is essential in laying the groundwork for biobehavioural synchrony *via* biopsychosocial mechanisms (Figures 2, 3). Furthermore, we suggest that touch can be used to establish and update priors in exchanges between parent and infant and between the practitioner and the infant. The intentional therapeutic touch delivered by a practitioner can promote allostatic regulation and establish a successful therapeutic alliance. Additional research on the quantifiable effects of therapeutic touch in children may better understand the mechanisms underlying already established treatments involving therapeutic affective touch. Furthermore, the effects of touch on reducing and preventing the development of maladaptive pain beliefs and pain itself may significantly enhance the effects of therapeutic affective touch.

## Data Availability

The original contributions presented in the study are included in the article/Supplementary Material, further inquiries can be directed to the corresponding author.
